# Pure red cell aplasia and amegakaryocytic thrombocytopenia in thymoma: The uncharted territory

**DOI:** 10.1002/ccr3.2642

**Published:** 2020-03-17

**Authors:** Matteo Dragani, Giacomo Andreani, Ubaldo Familiari, Valerio Marci, Giovanna Rege‐Cambrin

**Affiliations:** ^1^ Azienda Ospedaliero Universitaria San Luigi Gonzaga Università di Torino Orbassano Italy

**Keywords:** amegakaryocytic thrombocytopenia, aplasia, eltrombopag, T‐cell immunity, thymoma

## Abstract

Association between thymoma and pure red cell aplasia is already well‐documented in literature whereas acquired amegakaryocytic thrombocytopenia is rarely reported. In this case, even with the addition of eltrombopag to standard immunosuppression, the cytopenias did not improve, probably due to the lack of surgical resection of the tumor.

## Graphical abstract

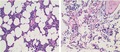

## 1 CASE PRESENTATION

A 62‐year‐old male was first diagnosed with lymphocyte‐rich thymoma in 2014 after being admitted in the emergency ward for sudden dyspnea and deep vein thrombosis of the right arm. His past medical history was uneventful, and he was nonsmoker. Notably, his blood examinations were perfectly normal. In June 2014, he was administered five cycles of chemotherapy based on standard CAPP (cisplatin, doxorubicin, cyclophosphamide, and prednisone) regimen obtaining a partial response.

From 2014 to 2017, the disease remained stable; in May 2017, the tumor burden increased with enlargement of the main mediastinal lesion and metastasis localized to the pleura, the diaphragm, and the second rib. In June 2017, the oncologist picked the CAPP regimen for a rechallenge but only one course of chemo was administered due to the appearance of normocytic anemia (Hb, 7 g/dL). At first, erythropoietin was prescribed but it did not show any effect.

The patient was refereed to our hematology department for further investigation. His anemia was not carential, not due to blood loss, not hemolytic, so prescription of erythropoiesis‐stimulating factor to contain chemotherapy‐induced anemia can be of help. Differential diagnosis includes performing a bone marrow biopsy to exclude therapy‐related myelodysplasia which can impair blood count recovery and led to aggressive forms of acute myeloid leukemia.

In July 2017, while the patient already has been put under blood transfusion weekly, a bone marrow biopsy was done, and it showed pure red cell aplasia (PRCA) with normal granulocyte maturation and megakaryocytes representation and a global cellularity around 50%. No cytogenetic abnormalities associated with myelodysplasia could be detected with FISH analysis, and remarkably no sign of thymoma metastasis in the marrow had been noticed. Blood count was as follows: WBC, 5900/μL; Hb, 6.5 g/dL; and platelets 327 000/μL.

Thymoma is the most common neoplastic lesion of the anterior mediastinum whose paraneoplastic complications such as myasthenia gravis (MG) and pure red cell aplasia (PRCA) are well‐known. About 25%‐30% of patients with thymoma experiences MG whereas approximately 2%‐5% of thymoma patients have pure red cell aplasia^1^ with the possibility of achieving complete remission of the latter after cyclosporine‐based immunosuppressive therapy, especially if total thymectomy is performed.

**Figure 1:**
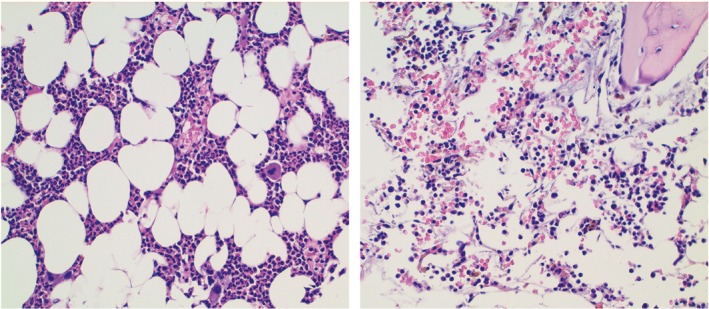
Bone marrow in July 2017 with absence of erythroid precursors, normal granulocytic, and megakaryocytic representation, cellularity around 50% (left); bone marrow in August 2017 with absence of both erythroid and megakaryocytic precursors, cellularity around 30% (right)

In August 2017, the patient developed severe thrombocytopenia with mucocutaneous bleeding (platelets: 14 000/μL). A second bone marrow biopsy was done exactly one month after the first one, and it showed pure red aplasia with amegakaryocytic thrombocytopenia (Figure 1). Cellularity was about 30% with normal granulocyte representation. Notably, no myeloid blasts were detected; WT1 rate was 20 and cytogenetic testing showed no abnormalities.

The development of severe thrombocytopenia just one month after the first biopsy where megakaryopoiesis showed no sign of impairment was unexpected. Patients with thymoma can experience thrombocytopenia in the context of aplastic anemia or immune thrombocytopenic purpura; in this case, aplastic anemia was ruled out due to the presence of 30% of cellularity with no sign of granulocytes’ alterations; immune thrombocytopenic purpura was excluded because it is associated with normal or increased megakaryocytes as a compensatory mechanism.

After August 2017, the patient was both red blood cell and platelets transfusion‐dependent. Combination therapy with cyclosporine 3 mg/kg, prednisone 50 mg/die, and dose‐escalation eltrombopag until the maximum dosage of 150 mg/die was promptly initiated with the addition of deferasirox as iron chelator. Four months of this combination therapy, from October 2017 to January 2018, did not provide any benefit.

The idea behind this therapy was that this step‐by‐step cytopenia could benefit by the combination of immunosuppression with a stimulating agent like eltrombopag, plus the addition of deferasirox which in some hematological malignancies has been able to cause erythroid response.^2^ Complete resection of the tumor was ruled out because of the metastasis and lack of a clear cleavage plan of the main lesion. Another course of chemotherapy was taken into consideration but excluded at the end to not further impair patient's performance status and to not risk pancytopenia and neutropenic fever.

At the end of January, for the first time a neutrophil count < 1.000/uL was observed. During these months of immunosuppressive therapy, thymoma remained stable but the patient was admitted twice in the emergency ward for sepsis. When the PRCA plus AT progressed to overt severe aplastic anemia, we interrupted eltrombopag and admitted the patients into the intensive hematological care unit to try a course of horse antithymocyte globulin (ATG) plus cyclosporine and high dose of steroids. Bone marrow transplantation from matched unrelated donor was taken into consideration as a salvage plan if the blood count would have shown no response to T‐lymphocyte depletion. Unfortunately, the patient died just before the ATG administration for a massive intracranial hemorrhage, despite multiple platelet transfusions and careful management of his blood pressure.

## 2 DISCUSSION

Only six patients have been reported in literature to have thymoma associated with pure red cell aplasia and amegakaryocytic thrombocytopenia (Table 1) with, as shown, different bone marrow characteristics and experiencing different oncologic management, from complete resection at first to standard chemotherapy (which includes cyclophosphamide, cisplatin, steroids, and doxorubicin).

**Table 1:** Case reports available on PubMed

Reports	Sex/Age	BM Evaluation	PRCA—AT timeline	Thymoma history
Maslovsky (2005)	M, 41 y	Pure red cell aplasia, absence of megakaryocytes, normal maturation and differentiation of myeloid precursors	PRCA first followed by AT after 3 mo	Thymoma diagnosed with PRCA; AT occurred after partial tumor resection and chemo.
Simkins (2017)	F, 61 y	BM cellularity 40%, erythroid and megakaryocytic aplasia with left‐shifted myeloid maturation	PRCA and AT at the same time	Lymphocyte‐rich thymoma, CAPP chemotherapy, radical thymectomy performed before PRCA and AT
Dahal (2018)	M, 60 y	BM hypercellular with severely depleted megakaryocytes and erythroid precursor cells and relative myeloid hyperplasia	PRCA and AT at the same time	Recurrent invasive thymoma, no surgery ever performed, diagnosis made before PRCA and AT
Onuki (2016)	F, 67 y	BM hypoplastic, decreased erythroid cells, scarce megakaryocytes, adequate number of myeloid cells	PRCA and AT at the same time	Thymoma occurred at presentation with PRCA and AT
Gay (2014)	M, 31 y	BM cellularity 20%, erythroid precursors 1%, markedly reduced to absent megakaryocytes	PRCA and AT at the same time	Relapsed thymoma after complete resection; PRCA and AT diagnosed after CAPP chemotherapy and partial remission
Fujiwara (2015)	F, 44 y	BM hypocellular with marked decrease in megakaryocytes and reticulocytes	PRCA first followed by AT (no time frame available)	Refractory thymoma diagnosed with PRCA, AT occurred after CAPP‐like chemotherapy and II line treatment with CAMP regimen
Present case (2018)	M, 62 y	BM cellularity 30%, normal granulocyte maturation, no evidence of erythroid and megakaryocytic precursors	PRCA first followed by AT after 1 mo	Refractory thymoma; no surgery ever performed; PRCA and then AT occurred after chemotherapy (first CAPP course, then rechallenge two years later).

Features of bone marrow, time frame of PRCA (pure red cell aplasia) and AT (amegakaryocytic thrombocytopenia) occurrence, and history of thymoma are shown.

Treatment in this series of patient always included cyclosporine backbone, alone or in combination with prednisone, and in two cases also horse ATG were administered. Eltrombopag has been used, beside our patient, in only one case but no improvement of platelet count was obtained; at the end a bone marrow, transplant from matched unrelated donor was successfully performed.^3^ Our case is an example where the addition of a TPO agonist did not provide an hematological recovery, whereas in the latest years this molecule has been proven effective in the treatment of severe aplastic anemia (SAA) both as a single agent in SAA refractory to IST or in frontline combination with cyclosporine/horse ATG.^4^, ^5^

Between these cases, four patients were able to improve their cytopenias^6^, ^7^, ^8^, ^9^ whereas in the remaining two cases information about follow‐up were not available and one patient left the hospital few days after therapy initiation.^9^, ^10^ Notably, in three cases including ours PRCA and amegakaryocytic thrombocytopenia (AT) progressed to aplastic anemia despite immunosuppression, thus empowering the theory, previously cited in the other reports, that PRCA and AT may be early presentation of complete aplasia.

Our impression is that patients with PRCA and AT with thymoma have better chances to achieve hematological improvement of their cytopenias if complete resection of the tumor can be performed at some point. Previous experiences indicate that both humoral and T cell–mediated immunity can be accounted as culprits of megakaryocytes suppression, and since thymomas can dysregulate both pathways we may assume that the presence of the tumor can overcome the effects of immunosuppressive therapy.^11^, ^12^ Nevertheless, certainties in this particular field are very scarce since thymoma itself is a rare entity and the discussion can only rely on sporadic experiences: further reports are needed as well as prospective studies to determine the role of eltrombopag, bone marrow transplantation, and surgical resection of the tumor.

## CONFLICTS OF INTEREST

The authors declare no conflicts of interest.

## AUTHOR CONTRIBUTIONS

MD, GRC, and GA: treated the patient. VM and UF performed the histological analysis. MD, GRC, and GA: wrote the paper.
